# The Embryology and Surgery of Double Ureter and Kidney

**Published:** 1917-05

**Authors:** Hugh Hampton Young, E. G. Davis


### The Embryology and Surgery of Double Ureter and Kidney.

BY HUGH HAMPTON YOUNG AND E. G. DAVIS.

The upper calculus pyonephrotic portion of left double kidney with a bifid ureter, in a man of 57, was diagnosed by means of pyelography, and the diseased portion successfully resected, leaving in situ the lower normal portion with undisturbed blood supply and ureter. The patient's symptoms, pain in the back and frequency of urination, were entirely relieved, and it was later demonstrated that the half kidney secreted normal urine.

A complete survey of the literature shows that bifid ureter and double kidney is surprisingly common, especially in autopsy records, and occurs in about 3 per cent of all individuals. In the surgical literature, however, only 26 case reports of this anomally could be found, and, of these, in only one (Albarran) was the diseased portion resected and the normal portion undisturbed. Since Albarran's report is very brief (only a few lines) and incomplete, the above case is apparently unique in that it is the only one fully reported.

As to the embryology of bifid ureter, there can be little doubt that this anomaly occurs as a result of a premature or exaggerated bifurcation of the tip of the ureteral bud, which bifurcation normally takes place to form the primitive renal pelvis shortly after the ureter buds off from the Wolffian duct. The ureteral bud appears, at about the 5 mm. stage, as an evagination from the caudal portion of the Wolffian duct, close to the union of the latter with the colaca. Complete double ureter is probably formed, either as the result of two separate evaginations from the Wolffian duct, or as the result of the process of dilatation of the lower end of the Wolffian duct (and of the uncleft lower end of the ureter) by which the former comes to form a part of the bladder wall.—*The Journal of Urology.*

